# Fatty acid recognition in the Frizzled receptor family

**DOI:** 10.1074/jbc.REV118.005205

**Published:** 2018-12-10

**Authors:** Aaron H. Nile, Rami N. Hannoush

**Affiliations:** From the Department of Early Discovery Biochemistry, Genentech, South San Francisco, California 94080

**Keywords:** fatty acid, Wnt signaling, lipid–protein interaction, dimerization, stem cells, cancer, G protein–coupled receptor (GPCR), cell signaling, binding, cysteine-rich domain (CRD), frizzled (FZD), glycoprotein, lipid-binding protein, glycoprotein

## Abstract

Wnt signaling regulates physiological processes ranging from cell differentiation to bone formation. Dysregulation of Wnt signaling is linked to several human ailments, including colorectal, pancreatic, and breast cancers. As such, modulation of this pathway has been an attractive strategy for therapeutic development of anticancer agents. Since the discovery of Wnt proteins more than 35 years ago, research efforts continue to focus on understanding the biochemistry of their molecular interactions and their biological functions. Wnt is a secreted glycoprotein covalently modified with a *cis-*unsaturated fatty acyl group at a conserved serine residue, and this modification is required for Wnt secretion and activity. To initiate signaling, Wnt proteins bind to cell-surface Frizzled (FZD) receptors, but the molecular basis for recognition of Wnt's fatty acyl moiety by the extracellular cysteine-rich domain of FZD has become clear only very recently. Here, we review the most recent developments in the field, focusing on structural and biochemical studies of the FZD receptor family and highlighting new insights into their molecular arrangement and mode of regulation by *cis*-unsaturated fatty acids. Additionally, we examine how other lipid-binding proteins recognize fatty acyl chains on Wnt proteins in the regulation of Wnt secretion and activities. Altogether, this perspective expands our understanding of fatty acid–protein interactions in the FZD system and provides a basis for guiding future research in the field.

## Introduction

The Wnt pathway has garnered attention because of its role in embryonic development, stem cell maintenance, and link to human disease. Mammals express 19 Wnt ligands that comprise secreted post-translationally modified glycoproteins with a *cis*-Δ9-unsaturated fatty acyl (palmitoleic acid, C16:1*n*-7) on a conserved serine residue (Ser-209 on Wnt3a) (1, 2). Palmitoleoyl is the predominant covalent modification on Wnt3a, although myristoleoyl (C14:1*n*-5) has also been observed, albeit at lower abundance (3). The fatty acyl modification of Wnt proteins is a tightly regulated process, leading to these proteins' proper secretion and activity (1). For instance, mutation of the fatty acylation site (Ser-209 in Wnt3a or Ser-224 in Wnt1) prevents the secretion of Wnt in cells (4–6).

Fatty acylation of Wnt proteins is catalyzed by the *O-*acyltransferase PORCN that utilizes a dedicated pool of unsaturated fatty acyl-CoAs, generated upstream through the action of the enzyme stearoyl-CoA desaturase SCD1 (4, 7). Labeling experiments utilizing alkyne (4) or radioactive fatty acids (8) demonstrated the incorporation of only a subset of fatty acids, 14–16 carbons in length, onto Wnt proteins by PORCN (4, 7, 9–11). Proper fatty acylation of Wnt is also required for its recognition by the co-chaperone Wntless, and abrogation of Wntless disrupts the secretion of Wnt proteins (12–17). Once Wnt proteins are secreted, their fatty acyl moiety is also critical for mediating their interaction with FZD^2^ receptors to initiate signaling. In the extracellular space, inactivation of Wnt ligands could occur via cleavage of their fatty acyl group by the secreted deacylase Notum. Conversely, ablation of Notum results in overactivation of Wnt signaling (18, 19). In sum, the fatty acyl modification on Wnt proteins is critical for their secretion and proper function through FZD receptors.

Frizzled receptors play diverse roles in physiological processes, including cell differentiation, bone growth, and stem cell regulation. Mammalian cells contain 10 FZD receptors that are clustered into four subfamilies based on their protein sequence homology: FZD1/2/7, FZD5/8, FZD3/6, and FZD4/9/10. FZD7 is integral in intestinal stem cell biology, as FZD7 knockout mice display impaired regeneration of their intestinal epithelium upon irradiating radiation (20). Consistent with this observation, treatment of intestinal organoids with FZD7 subtype–specific peptide antagonist (dFz7-21) (21) disrupts their morphology and impairs stem cell renewal.

FZD7 receptors also play a role in the pathogenesis of *Clostridium difficile*, an opportunistic bacterium that colonizes the human colon when native microbiota is disrupted, leading to a range of symptoms, including mild diarrhea to life-threatening inflammation of the colon. The TcdB toxin from *C. difficile* targets preferentially the FZD7 receptor subtype in order to facilitate toxin entry while simultaneously blocking Wnt-mediated intestinal stem cell self-renewal (22). Interestingly, full-length TcdB mutants defective in FZD binding display diminished capacity to disrupt the intestinal epithelium of WT mouse cecum compared with WT TcdB controls (23).

Moreover, several FZD receptors have been implicated in the progression of different cancers (24). For instance, treatment with anti-FZD5/8 antibodies inhibits the growth of RNF43-mutant pancreatic ductal adenocarcinoma cells (25). Unlike FZD5 and -7, FZD4 interacts with the extracellular regulator Norrin to stimulate Wnt signaling (26–28). In humans, blindness due to familial exudative vitreoretinopathy is associated with loss-of-function mutations in FZD4 or Norrin (29). Deletion of either FZD4 or Norrin in mice results in a similar phenotype, including vascular defects in the retina, cerebellum, and inner ear (27, 30).

Even though the molecular details for how FZD receptors function at the cell surface remain elusive, recent studies have started to shed new molecular insights onto the architecture of the extracellular CRD of these proteins. The FZD CRD comprises ∼120 amino acids, including 10 conserved cysteine residues that form disulfide bonds, and it exhibits a compact globular structure that is primarily composed of α-helical secondary structures with a prominent hydrophobic groove. In 2001, the first X-ray crystal structure of a member of the FZD family, mouse (m) FZD8 CRD, was reported (31). Subsequently, the X-ray crystal structures of FZD4, FZD5, FZD7, and FZD2 CRDs were reported between 2015 and 2018, revealing a conserved fold among all these FZD CRDs (23, 32–35), with some variations noted in the overall dimer architecture of the CRD, in particular for FZD4 CRD. In this perspective, we review the recent biochemical and structural data on the architecture of FZD CRDs and their regulation by fatty acids. We focus on the molecular aspects governing lipid–protein interactions, in particular highlighting the recognition of fatty acyls by the lipid-binding cavity present on different FZD protein family members.

## Structure of XWnt8–FZD8 CRD complex

**Figure 1.**
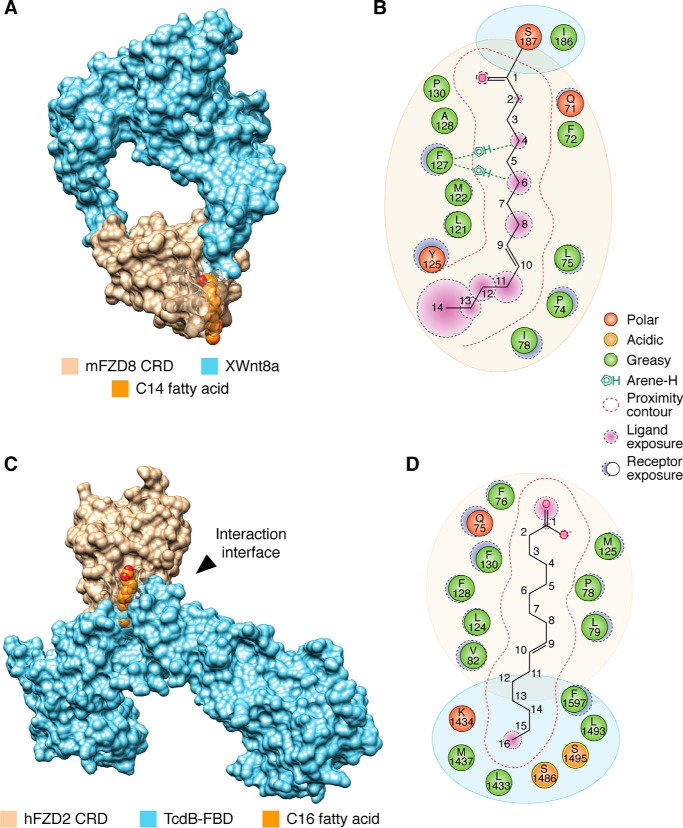
**Fatty acids contribute to Frizzled protein–protein interactions.** *A*, crystal structure of XWnt8a in complex with mFZD8 CRD (surface representation; PDB code 4F0A) highlighting the C14 fatty acid covalently attached to XWnt8a that occupies the hydrophobic groove of mFZD8 CRD with (*B*) the interaction map of the fatty acid–binding site. *C*, crystal structure of FZD2 CRD (surface representation; *tan*) in complex with the FBD of TcdB (*blue*, surface representation; PDB code 6C0B) bridged by a C16 fatty acid (*orange,* space-filling model), with (*D*) interaction map of fatty acid–binding site. The saturation status of each fatty acid could not be determined due to the resolution of the structure. Artwork by Luciana Giono.

The first X-ray crystal structure of a Wnt protein bound to FZD CRD was reported in 2012. It was demonstrated that *Xenopus* (X) Wnt8a interacted with mFZD8 CRD (1:1 stoichiometry) at two distinct sites, one of which comprised a lipid–protein interaction. In the lipid-binding cavity, an electron density was observed that corresponds to a fatty acyl group emanating from Ser-209 of XWnt8a (Fig. 1, *A* and *B*) (34). Most of the hydrocarbon chain was buried within the hydrophobic groove. However, some details surrounding that nature of the fatty acid–protein interaction interface remained elusive. For instance, the degree of unsaturation and length of the hydrocarbon chain on XWnt8 was inconclusive (34) and hence did not provide molecular insights into how a *cis*-unsaturated fatty acid might be accommodated within the hydrophobic groove of FZD CRD. Another unanswered question pertained to the protrusion of the last few carbon atoms at the methyl end outside the hydrophobic groove. The protruding lipid tail is shielded by a neighboring XWnt8 molecule in the crystal lattice (referred to as site 3), thereby precluding any possible CRD dimerization. Finally, it is noteworthy that the observed monomeric state of the mFZD8 CRD is in contrast to the dimer form that has been frequently observed in solution and several reported X-ray crystal structures (35, 36), suggesting that the CRD monomer state captured in the XWnt8–mFZD8 CRD complex could be a precursor or intermediate to a pre-formed dimer (see below).

## Fatty acid bridging as a mechanism for FZD–CRD interaction with toxins

TcdB from *C. difficile* hijacks FZD1, -2, and -7 receptors for toxin entry. The crystal structure of FZD2 CRD was recently solved in complex with TcdB frizzled-binding domain (FBD; residues 1285–1804) to 2.5 Å resolution (Fig. 1, *C* and *D*) (23). In the crystallographic model, FZD2 CRD and TcdB form a T-shaped complex in which the CRD constitutes one of the T arms (Fig. 1*C*). A 16-Å–long tubular electron density connects TcdB to FZD2 CRD. Interestingly, this arrangement is reminiscent of the crystal packing at site 3 observed in the XWnt8a–mFZD8 CRD structure (34), although site-directed mutagenesis of site 3 yielded mixed results about its functional relevance in a TCF4/Wnt firefly luciferase reporter (37), suggesting that it may be a crystallographic artifact.

Because of insufficient resolution, the authors could not determine the degree of unsaturation of the fatty acid in the TcdB–FZD2–CRD complex, although it was designated as *cis*-C16:1*n*-7. The interaction between the fatty acid and the lipid-binding cavity of FZD2 CRD is primarily mediated through hydrophobic interactions. The carboxylic acid headgroup is in close proximity to Gln-75, Phe-76, Met-125, and Phe-130 residues on FZD2 CRD. The middle section of the hydrocarbon chain is stabilized by both the CRD and TcdB, including residues Pro-78, Val-82, Leu-79, and Leu-124 on the CRD and Phe-1597 on TcdB (Fig. 1*D*). The ω-carbons are positioned near Tcdb residues Leu-1433, Met-1437, Ser-1486, Leu-1493, and Ser-1495, in a pocket that is formed at the interface between the CRD and TcdB, thereby shielding the fatty acid from the aqueous environment. The structural observations are supported by biophysical and biochemical data demonstrating that the addition of fatty acid promoted the interaction between FZD5 CRD and TcdB (23). Moreover, TcdB mutations predicted to disrupt fatty acid binding and/or FZD2 CRD interactions disrupted the complex (23). Interestingly, mutations in the lipid-binding cavity of FZD2 CRD (*e.g.* F76A, F76D, L79D, and M125D) led to glycosylation and secretion defects, suggesting that intracellular fatty acid binding may be physiologically important for the proper folding, secretion, and glycosylation of FZD2 CRD in HeLa cells (23). In sum, these data highlight a role for fatty acids in mediating the recognition of FZD CRDs by TcdB and a possible function as chaperones for FZD receptors. Deeper investigation into the identity of fatty acids, which can mediate the interaction, is warranted, as multiple fatty acid species could promote this interaction.

## Molecular characteristics of the fatty acid–binding groove in FZD receptors

### U-shaped lipid–binding cavity within dimeric FZD5 and FZD7 CRDs

**Figure 2.**
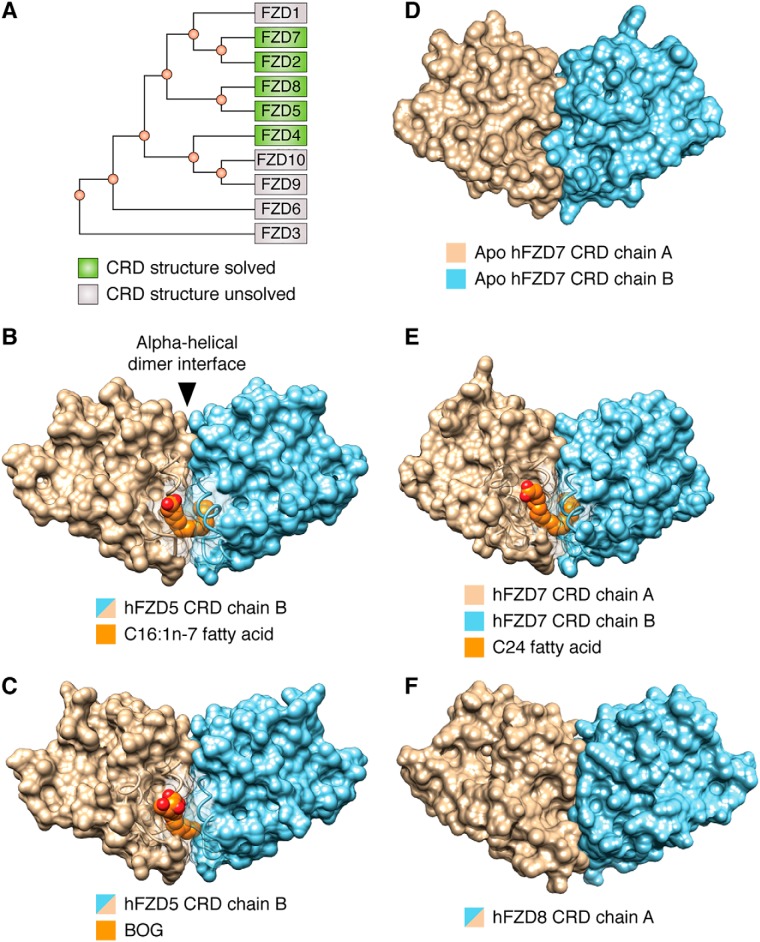
**FZD5 and FZD7 subclass CRDs share a conserved α-helical dimer interface and U-shaped lipid-binding cavity.** *A*, phylogenetic tree of hFZD CRDs. Surface representations of hFZD5 CRD in complex with C16:1*n*-7 fatty acid (PDB code 5URY) (*B*), hFZD5 CRD in complex with BOG (PDB code 5URZ) (*C*), apo hFZD7 CRD (PDB code 5T44) (*D*), or in complex with C24 fatty acid (PDB code 5URV) (*E*), and apo mFZD8 CRD (PDB code 1IJY) (*F*) are shown. Each hFZD5 CRD ligand represents 50% occupancy due to their location along the 2-fold symmetry axis and only one molecule is displayed for clarity. Surfaces within ∼7 Å of the ligand are transparent. The N terminus of hFZD7 CRD in complex with C24 fatty acid is truncated for visual clarity. Artwork by Luciana Giono.

The first structures of FZD CRDs bound to a free fatty acid were reported in 2017, as exemplified by human (h) FZD5 CRD in complex with *cis*-C16:1*n*-7 fatty acid or with *n*-octyl-β-d-glucoside (BOG) (Fig. 2, *A–C*) (35). The fatty acid served as a surrogate for the Wnt fatty acyl group. In both structures, FZD CRD crystallized as a dimer, comprising a previously uncharacterized α-helical dimer interface (35). Consistent with these findings, the first reported X-ray crystal structures of FZD7 CRD in either its apo or C24 fatty acid-bound form (Fig. 2, *D* and *E*) revealed a strikingly similar CRD dimer arrangement (35), with an α-helical dimer interface as that observed in FZD5 CRD.

In support of the above observations, FZD8 CRD also exhibited a dimeric arrangement with an α-helical dimer interface and a U-shaped lipid-binding cavity (Fig. 2*F*) (35), similar to that of FZD5 and FZD7 CRDs in the lipid-bound or nonbound states. It is noteworthy that the initial X-ray crystal structure report of mFZD8 CRD highlighted a dimer configuration with a loop–loop interface as the asymmetric unit, based on similarity to the then known soluble Frizzled-related protein 3 (sFRP3) loop-loop dimer interface (31). However, a later study revealed that the same mFZD8 CRD structure also comprised an α-helical dimer interface (35). As a result, the biological unit was revisited based on *in silico* energetics that favored the α-helical dimer interface configuration. In this context, the binding mode of the fatty acid is energetically favored because the hydrocarbon chain is fully buried within the U-shaped hydrophobic cavity and shielded from solvent.

**Figure 3.**
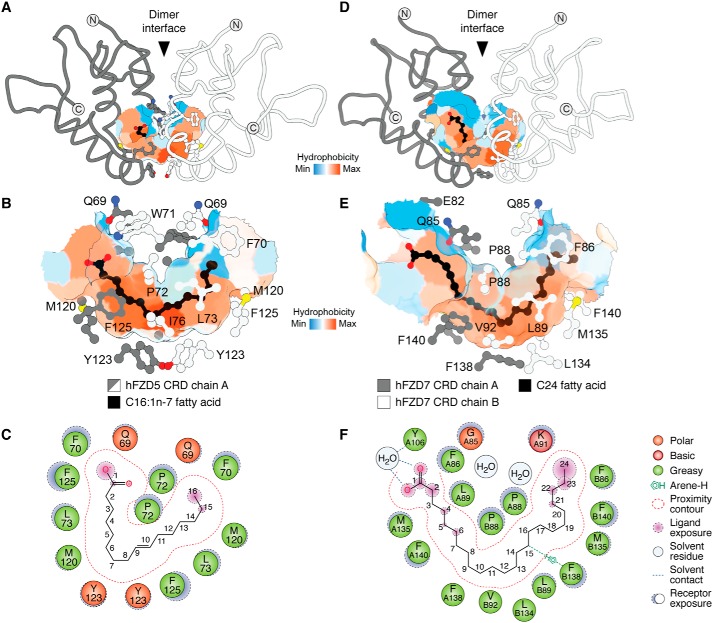
**Structural basis for fatty acid recognition by Frizzled CRDs.** Surface representations of the hydrophobic cavity of hFZD5 CRD in complex with C16:1*n*-7 (*A*) with a zoomed-in view without polypeptide backbone highlighting residues proximal to the hydrophobic cavity (*B*) are shown. *C*, ligand interaction map illustrating interactions between hFZD5 CRD and C16:1*n*-7 (PDB code 5URY). Surface representations of the hydrophobic cavity in the C24-bound hFZD7 CRD structure (*D*) with a zoomed-in view (PDB code 5URV) (*E*) are shown. *F*, ligand interaction map between hFZD7 and C24 fatty acid. Fatty acid carbons are numbered sequentially, starting from the carboxylic acid headgroup (C1). Artwork by Luciana Giono.

In the FZD5/7/8 CRD structures, the two hydrophobic grooves present on each monomer converge at the dimer interface, thereby creating a contiguous U-shaped hydrophobic cavity that buries the BOG or C16:1*n*-7 ligands (Fig. 3) (35). The contour of the hydrophobic cavity in FZD5 CRD bound to C16:1*n*-7 is formed, in part, by residues Phe-125, Leu-73, Met-120, Tyr-123, and Pro-72, with carbons 9–10 (representing the unsaturation site of the fatty acid) positioned at the base of the cavity (Fig. 3, *B* and *C*). The contour of the hydrophobic cavity in FZD7 CRD bound to C24 is formed, in part, by Phe-86, Pro-88, Leu-89, Val-92, Met-135, Leu-134, Phe-138, and Phe-140, with carbon atoms 9–12 positioned at the base of the cavity (Fig. 3, *E* and *F*).

### FZD4 CRD adopts multiple dimer configurations

**Figure 4.**
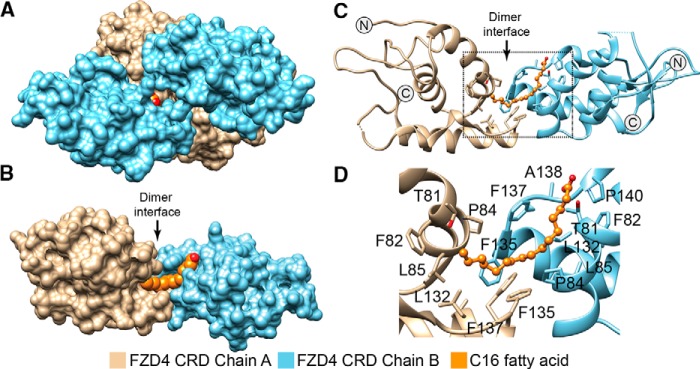
**FZD4 CRD binds free fatty acids at its hydrophobic cavity.** *A*, crystal structure of tetrameric FZD4 CRD (surface representation) in complex with two 16-carbon fatty acids (space-filling model, *orange*; PDB code 5UWG). Only the carboxylic headgroup of one fatty acid is visible. *B*, surface representation of the chain A-B dimer from *A. C*, ribbon representation of FZD4 CRD from highlighting residues within ∼5 Å of the C16 fatty acid (*B*) with a zoomed-in view (*D*). Glycans and water are hidden for clarity. The saturation status of the fatty acid could not be determined due to the resolution of the structure.

Unlike the FZD5/7/8 CRD structures, the initial structures of the FZD4 CRD dimer, including its apo-form (32) or in complex with extracellular regulator Norrin (32, 33), showed that it crystallized into multiple configurations that lacked an α-helical dimer interface. However, the recently reported studies of FZD4 CRD in complex with a free fatty acid (solved to 2.56 Å resolution; PDB code 5UWG) (36) comprised a dimer with an interface formed in part by residues Gln-87, Pro-84, Tyr-88, Val-131, Phe-137, Phe-135, and Gln-134 on each monomer; these residues include several of the cognate residues found on the FZD5/7 CRD α-helical dimer interface (Fig. 4).

Similar to what was observed in the FZD5 and FZD7 CRD structures, the C16 fatty acid in FZD4 CRD structure traverses the dimer interface making contact with both monomers (Fig. 4, *C* and *D*) (36). It is noteworthy that the electron density is not well defined in the structure. The carboxylic acid headgroup of C16 is in close proximity to Pro-140, Phe-137, and Phe-82. Further down the groove, the midsection of the hydrocarbon chain is supported by Pro-84 and Phe-135, whereas the methyl end comes into contact with Leu-132 and Leu-85. Finally, there is another chain A-B FZD4 CRD dimer that is observed in the asymmetric unit, leading to a tetrameric FZD4 CRD state and forcing the hydrophobic groove to an extended state (Fig. 4*A*). Yet, the biological relevance of the tetramer is not clear at this point, and its occurrence seems unlikely given the significant clashes that will occur upon Wnt binding (based on superimposition with XWnt8a–mFZD8 CRD co-structure, PDB code 4F0A; data not shown).

**Figure 5.**
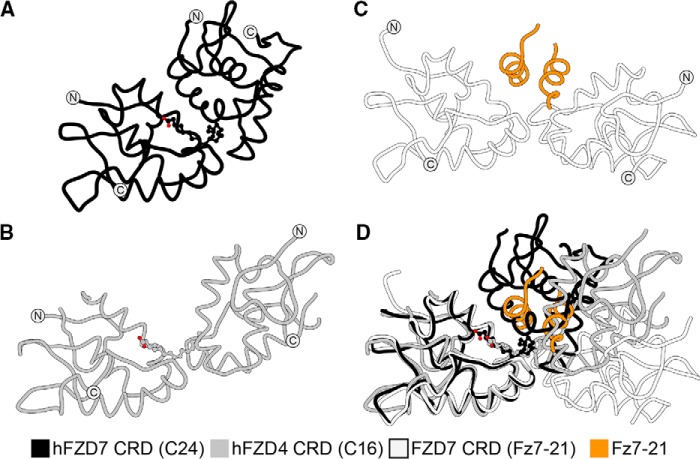
**FZD CRD dimers exist in multiple conformations.** Structural representations of human hFZD7 CRD in complex with C24 fatty acid (PDB code 5URV) (*A*), FZD4 CRD (chain A–B) in complex with C16 fatty acid (PDB code 5UWG) (*B*), hFZD7 CRD bound to the antagonistic dFz7-21 peptide (*orange*) (*C*), and superimposition of all structures (*D*) are shown.

Overall, the architecture of the FZD4 CRD dimer seems similar to that of FZD7 CRD but with some notable variations, in particular the broader spacing between the monomers at the α-helical dimer interface (Fig. 5). This “extended” conformation is reminiscent of a recent FZD7 CRD structure bound to a peptide antagonist dFz7-21 (21), in which the α-helical dimer interface is further extended and therefore represents a most extreme case of an extended CRD dimer conformation to date to our knowledge. When superimposed, these structures suggest that FZD CRDs might sample multiple conformations with a varying degree of flexibility or spacing around the α-helical dimer interface (21).

## FZD CRD dimerization

Collectively, the X-ray crystal structures suggest that FZD CRDs exist in multiple states, including dimer as well as monomer. In solution, recombinant purified FZD7 CRDs adopt dimer and monomer states, in addition to other unidentified states (31, 35). It has been demonstrated that FZD receptors can form homodimers when overexpressed on the cell surface, although the biological significance of this dimerization is still unclear. β-Gal complementation assays carried out on FZD1, FZD2, FZD4, FZD7, and FZD9 established the formation of homo-oligomers between these proteins (26). Pulldown experiments have further shown that FZDs and FZD-related proteins can dimerize (38, 39). In *Xenopus* embryos, FZD receptor dimerization seemed to correlate with activation of Wnt signaling (39). Additionally, bioluminescence resonance energy transfer assays showed that Wnt5A stimulates dimerization of membrane-anchored FZD4 CRD or full-length FZD4 (36). Moreover, single-molecule receptor intensity analysis in HeLa cells demonstrated that FZD8 CRD is primarily monomeric but transitions to dimeric, trimeric, and oligomeric species after the addition of XWnt8a or Wnt3a (40). In fact, additional dimer states might exist. For instance, peptide dFz7-21 revealed an FZD7 CRD trapped in a dimer conformation that is nonfunctional. All in all, the above results collectively suggest that FZD receptors are in equilibrium, sampling multiple states including preformed dimers and monomers, and a shift in their equilibrium states might regulate downstream outcomes in the presence and absence of Wnt ligands. The biochemical data suggest that that this equilibrium might be regulated by the receptor's local concentrations or the presence of Wnt ligand, among other factors.

The X-ray crystallographic data (35, 36) suggest that fatty acids may play a role in stabilizing the dimeric conformation of FZD CRDs. We demonstrated recently that the addition of free unsaturated fatty acids, at least C14 in length, promoted dimerization of FZD7 CRD in solution as measured by size-exclusion chromatography (35). It is remarkable that a shift to a dimer state could be detected in solution given that these studies were done under equilibrium conditions carried out with soluble CRDs in the absence of full-length receptor or plasma membrane that could aide in receptor clustering. Nonetheless, incubation of monomeric FZD7 CRD with saturating concentrations of fatty acids with variable length and degrees of unsaturation in their hydrocarbon chains showed that unsaturated fatty acids (*e.g.* C14:1, C16:1, C18:2, C18:3, C20:4, and C20:5) were more effective at enhancing CRD dimerization compared with their saturated counterparts (*e.g.* C8:0, C10:0, C12:0, C14:0, and C16:0). These data are consistent with the structural predictions about the need of at least C14 chains to bridge the FZD CRD dimer interface and promote dimerization (35). Although these biochemical studies are the first to reveal fatty acid-mediated dimerization of FZD CRDs, additional structural studies coupled with lipid biochemistry experiments will be needed to further characterize the biological role of the dimer. For instance, experiments aimed at understanding the dynamics of the α-helical dimer interface and its regulation of the U-shaped hydrophobic cavity such as hydrogen–deuterium exchange, NMR, crystallography, cross-linking, and more in-depth mutational analysis are needed to further validate the role of CRD dimerization in Wnt signaling.

Overall, the structural and biochemical data are consistent with a model in which a Wnt molecule could bind to an FZD CRD dimer, with the Wnt fatty acyl group promoting dimerization of FZD CRD. How this 1:2 Wnt/FZD arrangement regulates ternary complex assembly at the cell surface and subsequent downstream signaling requires further investigation.

## Recognition of fatty acids by FZD CRDs

The X-ray crystal structures of multiple FZD CRDs suggest that there are some common features and mechanisms mediating the recognition of the fatty acyl group, including (*a*) positioning of the fatty acyl chain along the contour of the lipid-binding cavity, with the unsaturation site residing at the base of the cavity; (*b*) a conserved set of residues that form the lipid-binding cavity; (*c*) notable flexibility in the side chains of residues outlining the cavity and their polypeptide backbone; and (*d*) inherent flexibility in the conformation of the fatty acyl chain. Collectively, these features, coupled with the overall dimeric architecture of the CRD, contribute to the recognition mechanism of fatty acids by FZD CRDs. Below, we provide a detailed discussion of these points.

### Positioning of the fatty acyl chain within the U-shaped hydrophobic cavity

**Figure 6.**
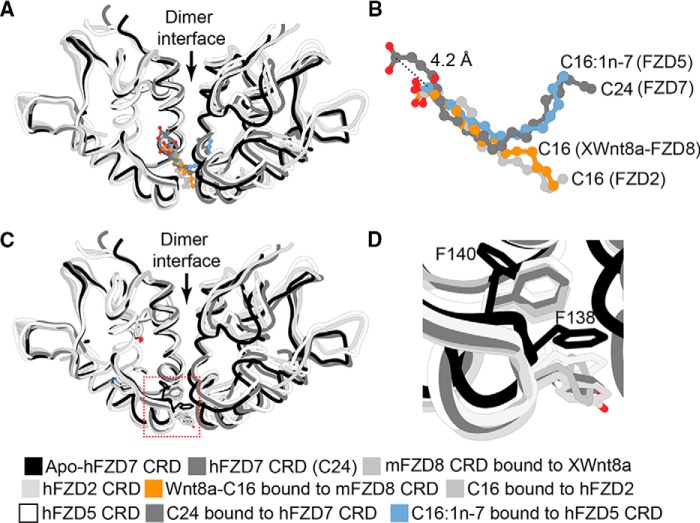
*A*, superimposition of apo hFZD7 CRD (PDB code 5T44), hFZD7 CRD in complex with C24 fatty acid (PDB code 5URV), apo mFZD8 CRD (PDB code 1IJY), hFZD2 CRD in complex with C16 fatty acid (PDB code 6C0B; FBD hidden for clarity), hFZD5 CRD in complex with C16:1*n*-7 fatty acid (PDB code 5URY), and mFZD8 CRD in complex with palmitoylated XWnt8a (PDB code 4F0A; XWnt8a protein hidden for clarity); with *B*, a zoomed-in view highlighting the fatty acids. *Dashed line* indicates distance from the carboxylic acid headgroup of C24 fatty acid to the carboxylic acid headgroup of C16:1*n*-7. *C*, structures from *a* highlighting the side chain conformations of residues Phe-138 and Phe-140 within the apo or lipid-bound FZD CRD structures, with *D*, a zoomed-in view. The side chains display an inward conformation that infiltrates the hydrophobic cavity in the apo FZD CRD structure but point away from the cavity when a fatty acid is present.

A high degree of similarity is observed in fatty acid recognition by FZD CRD when comparing the binding mode of C24 *versus* C16:1*n*-7 fatty acids (35). Both fatty acids occupy and follow the contour of the U-shaped hydrophobic cavity, traversing the α-helical dimer interface. However, the carboxylic acid within the longer C24 chain is positioned further up, away from the opening of the hydrophobic cavity and ∼4.2 Å distance from the C16:1*n*-7 carboxylic acid headgroup (Fig. 6, *A* and *B*). The carboxylic acid headgroup of C24 is coordinated through a water molecule to a conserved Tyr-106 near the entrance of the hydrophobic cavity (Fig. 6, *A* and *B*). In contrast, the carboxylic acid headgroup of C16:1*n*-7 is shifted down near Gln-69 at the entrance of the hydrophobic cavity (Fig. 6*B*). It is noteworthy that C16:1*n*-7 headgroup superimposes well with the ester group bridging the fatty acyl to Wnt protein in the structure of the *X*Wnt8–FZD8 CRD complex. These results suggest that the placement of the free acid C16:1*n*-7 is in a biologically relevant position (35).

The hFZD5 and FZD7 structures are not of sufficient resolution to further resolve differences in the C9–C10 *cis*-alkene, and therefore, higher-resolution structures would be required to further refine the geometry of the double bond and its recognition within the U-shaped hydrophobic cavity of FZD CRD. Similarly, the degree of unsaturation of the hydrocarbon chain within C24 could not be resolved. Nonetheless, there seems to be a preference for the positioning of the unsaturation site at the base of the cavity. These findings suggest that FZD CRDs exhibit plasticity within their lipid-binding cavities for recognition of fatty acids with different chain lengths.

### Plasticity in the fatty acyl-binding groove

Comparison between mFZD8 CRD bound to XWnt8a and apo mFZD8 CRD structures originally revealed that the fatty acyl binding groove did not display any obvious conformational changes in the presence and absence of a bound lipid (34). However, closer inspection of the structure of mFZD8 CRD revealed the presence of tubular electron densities within the hydrophobic cavity, suggesting the possibility of a bound ligand of unknown identity (35). Comparison of the structures of apo FZD7 CRD with those of fatty acid-bound FZD5, -7, and -8 CRDs indicates that there is considerable flexibility within the lipid-binding cavity (35). In particular, residues Phe-138 and Phe-140, which comprise a Phe–Gly–Phe motif (referred to later as the FGF motif) that lines the surface of the hydrophobic cavity, point inward and toward the lipid-binding cavity, and they occupy the lipid-binding cavity in the absence of a bound fatty acid (Fig. 6, *C* and *D*). In contrast, they display an outward orientation away from the cavity in the presence of a fatty acid bound in the cavity. Although the functional significance of this notable movement in the side-chain orientations of the FGF motif is not clear yet, it may provide a mechanism to regulate fatty acid binding and/or selectivity toward particular fatty acids. We hypothesize that plasticity within the hydrophobic cavity might account for binding to different fatty acids with varying degrees of unsaturation in their hydrocarbon chains.

In the Tcdb–FZD2 CRD complex structure, the C16 fatty acid follows a linear trajectory along the FZD2 CRD hydrophobic cavity, with a geometrical shape that is similar to what was observed for C16 fatty acid in the XWnt8a–mFZD8 CRD complex (PDB code 4F0A) (Fig. 6*B*) (34). It is also distinct from that seen in FZD5 CRD in complex with C16:1*n*-7 (Fig. 6*B*) (35). However, the side chains of residues lining the hydrophobic cavity in the FZD2 CRD–TcdB complex are pointing away from the cavity, consistent with the side chain orientations observed in other fatty acid-bound FZD CRD structures (Fig. 6*D*).

## Fatty acid recognition by other Wnt-regulating proteins

### Afamin

**Figure 7.**
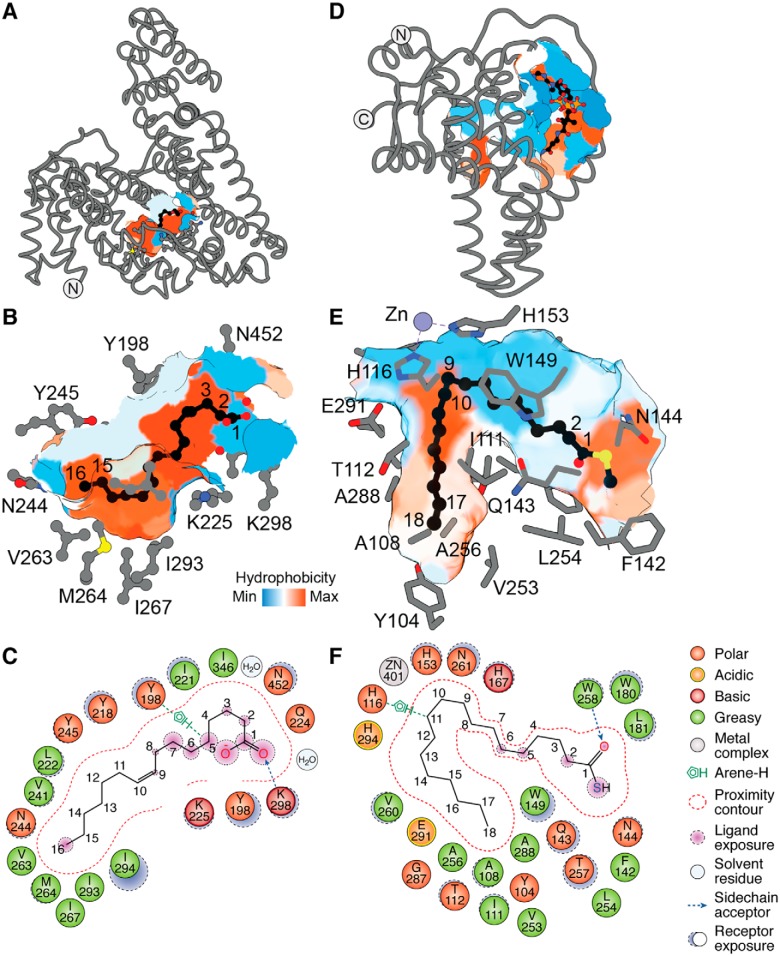
**Fatty acid-binding proteins utilize diverse cavities with specific geometry to accommodate their ligands.** *A*, structure of affamin in complex with C16:1*n*-7 fatty acid bound at its hydrophobic groove (surface representation), with *B*, a zoomed-in view highlighting side chains of residues within ∼5 Å distance from the fatty acid (PDB code 5OKL). *C*, ligand interaction map of C16:1*n*-7 in complex with affamin. *D*, structure of mSCD1 in complex with CoA–C18:0 occupying its hydrophobic cavity, with *E*, a zoomed-in view highlighting side chains of residues within ∼5 Å distance from the bound lipid chain (PDB code 4YMK). *F*, ligand interaction map of the fatty acyl moiety of the C18:0–CoA. The CoA moiety was hidden for clarity to highlight the acyl chain. Artwork by Luciana Giono.

Multiple mechanisms have been proposed to explain how the hydrophobic Wnt ligands might function in aqueous environments to avoid aggregation and nonspecific interactions with membranes (1). The chaperone Afamin, a glycoprotein component of blood serum, forms a 1:1 complex with Wnt3a and Wnt5a, thereby shielding the Wnt fatty acyl group and reducing its propensity for aggregation while still maintaining its bioactivity (41). Afamin is an 87-kDa protein that comprises a large hydrophobic cavity. It is structurally related to albumin that has been crystallized in at least 187 instances, three of which are in complex with palmitic acid (PDB codes 1E7H, 4BKE, and 4Z69). The structure of afamin was also solved recently in complex with a *cis*-C16:1*n*-7 fatty acid to 2.1 Å resolution (PDB code 5OKL) (Fig. 7, *A–C*) (42–44). The fatty acid binds in the hydrophobic cavity in a slightly spiraled fashion only partially occupying the hydrophobic cavity. The carboxylic acid headgroup is coordinated by Lys-298 through a hydrogen bond (Fig. 7, *A–C*). The hydrocarbon chain makes numerous hydrophobic interactions with Asn-452, Tyr-198, Ile-221, Ile-294, Leu-222, Met-264, and Asn-244, to name a few, within the hydrophobic cavity. In addition to the lipid–protein interactions, there are protein–protein interactions that mediate the binding between afamin and Wnt.

### Stearoyl-CoA desaturase

The Wnt fatty-acyltransferase porcupine utilizes a dedicated pool of *cis*-C16:1–CoA generated by the endoplasmic reticulum-resident sterol CoA desaturase, SCD1 (4, 7). Two independent groups crystallized human and mouse SCD1 membrane proteins in complex with stearoyl-CoA (PDB codes 4YMK and 4ZYO) (45, 46). The CoA moiety is solvent-exposed and makes a number of contacts on the surface through hydrophilic and charged residues (Fig. 7*D*). The fatty acyl chain threads through a hydrophobic cavity and, around half-way through, undergoes a dramatic change in direction toward the floor of the cavity (Fig. 7*E*). This change in orientation is critical for catalysis as it positions the 9th and 10th carbons of the chain in close proximity to the histidine box of SCD1 to undergo a regio-selective and stereoselective *syn* dehydrogenation reaction (Fig. 7, *E* and *F*).

All in all, it seems that nature has evolved lipid-binding cavities with specific geometrical shapes and constraints to recognize fatty acids in different configurations (1).

## Conclusion

The past few years have witnessed significant progress in the elucidation of frizzled CRD structure and function. Emerging structural data revealed a new FZD CRD dimer state bound to fatty acids, which challenge the status quo and the prevailing view about the architecture of FZD CRDs, suggesting new possibilities for the assembly of Wnt–FZD complexes. In a model based on biochemical and structural data, a single Wnt molecule could bind to a FZD CRD dimer and potentially help mediate dimerization of FZD receptors through its fatty acyl modification.

The new data also shed light onto different states that FZD CRDs adopt, suggesting that they exist in equilibrium between the monomer and dimer. Dimers also seem to exist in functional and nonfunctional conformations, the latter of which were revealed by utilizing a pharmacological peptide probe that traps a specific conformation of FZD CRD dimer (21). A Wnt ligand when presented to the surface is expected to perturb the receptor equilibrium to shift the population to a signaling-competent CRD conformation. We speculate that monomers and pre-formed dimers might exist on the cell surface, and Wnt ligands would enrich the population of these dimers via their fatty acyl groups, hence shifting the equilibrium to a fully active FZD receptor state that promotes clustering of downstream components and signalosome assembly (35).

The structural observations suggest new scientific hypotheses that could be tested and open up a world of experiments to address questions such as how fatty acids might regulate Wnt signaling and how they would influence FZD CRD interactions on the cell surface. In particular, are FZD CRDs intrinsically bound to fatty acids on the cell surface and, if so, what are their oligomeric states? Do fatty acids impact or elicit cross-talk between the CRD and the FZD transmembrane domain? Do intracellular fatty acids associate with FZDs and influence their protein secretion? In this case, could fatty acid metabolism be a regulator of FZD expression levels? Other questions pertain to the role of exogenous fatty acids and potentially our diet in regulating Wnt signaling and infections. *C. difficile* TcdB binds FZD2 CRD when the latter is primed with fatty acids, suggesting that a population of FZD receptors in *vivo* could be pre-bound to fatty acids. These observations also raise a question about how different FZD states signal at the cell surface. Understanding how the natural Wnt ligands influence FZD receptor equilibrium states on the cell surface remains critical for delineating FZD receptor signaling. In this regard, developing methods to monitor FZD clustering and visualize fatty acid association with FZD receptors in cells would be invaluable. Finally, ambiguity in the precise conformation of the fatty acid hydrocarbon chain will require high-resolution structures to further understand the nature of the fatty acid interactions within FZD CRD complexes. Remarkably, the first structure of an FZD transmembrane region without its CRD was recently reported (47), revealing new insights about the architecture of the transmembrane domain. This, along with the reported CRD structures, provides a molecular basis for understanding this receptor class, and it propels future efforts aimed at further mechanistic investigations of its function in cell signaling and disease biology.

## Footnotes

2 The abbreviations used are:

FZD: Frizzled
CRD: cysteine-rich domain
PDB: Protein Data Bank
h: human
m: mouse
X: *Xenopus*
FBD: frizzled-binding domain
BOG: *n*-octyl-β-d-glucoside.
